# Supplementary figures and images for: Genes for degradation and utilization of uronic acid-containing polysaccharides of a marine bacterium Catenovulum sp. CCB-QB4
Source: PeerJ. 2021 Mar 9;9:e10929. doi: 10.7717/peerj.10929 (PMC7953866; doi:10.7717/peerj.10929)

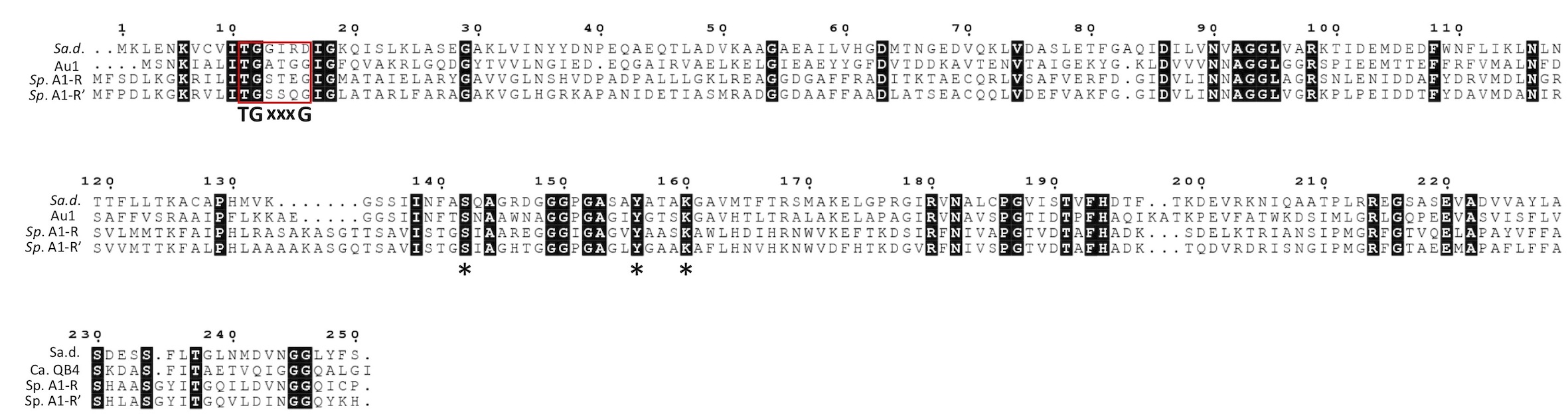

Supplement: Supplemental Information 1 — Red boxes indicate a TGXXXGX motif. Asterisks indicate catalytic triads of the enzyme. Sa.d., Saccharophagus degradans; Sp. Sphingomonas sp. The figure was drawn using the program ESPript (Robert and Gouet, 2014). Reference: Robert, X. and Gouet, P. (2014) ”Deciphering key features in protein structures with the new ENDscript server”. Nucleic. Acids Research 42 (W1), W320-W324 - doi: 10.1093/nar/gku316. [file peerj-09-10929-s001.jpg]
