# Supplementary material for: Genes for degradation and utilization of uronic acid-containing polysaccharides of a marine bacterium Catenovulum sp. CCB-QB4
Source: PeerJ. 2021 Mar 9;9:e10929. doi: 10.7717/peerj.10929 (PMC7953866; doi:10.7717/peerj.10929)
Supplement: Supplemental Information 2 [file peerj-09-10929-s002.docx]

Table S1. The similarities of amino acid (aa) sequence of alginate metabolic enzymes of QB4 in PDB database.

Name Description (PDB ID) aa sequence

similarity (%)

Ad1_PL6 Alygc, *Paraglaciecola chathamensis* (5GKD) 74.20

Ad2_PL6 AlyQ, *Persicobacter* sp. CCB-QB2 (5XNR) 71.60

Ad3_PL6 Alygc, *Paraglaciecola chathamensis* (5GKD) 48.90

Ad4_PL7 AlyQ, *Persicobacter* sp. CCB-QB2 (5XNR) 75.57

Ad5_PL7 AlyQ, *Persicobacter* sp. CCB-QB2 (5XNR) 71.17

Ad6_PL7 Alginate lyase, *Klebsiella pneumoniae* (4OZX) 49.70

Ad7_PL7 Alginate lyase, *Klebsiella pneumoniae* (4OZX) 71.27

Ad8_PL17 Alginate lyase, *Saccharophagus degradans* 2-40 (4NEI) 70.00

Au1 DEH reductase A1-R', Sphingomonas sp. A1 (4W7I) 53.94

Au2 KDG kinase, *Shigella flexneri* (3LHX) 64.05

Au3 KDG kinase, *Shigella flexneri* (3LHX) 54.49

Au4 2-keto-3-deoxy-6-phosphogluconate aldolase, 58.70

*Thermotoga maritima* (1WA3)

Au5 Kdpg aldolase, *Escherichia coli* (1EUA) 84.47
