# Supplementary material for: Genes for degradation and utilization of uronic acid-containing polysaccharides of a marine bacterium Catenovulum sp. CCB-QB4
Source: PeerJ. 2021 Mar 9;9:e10929. doi: 10.7717/peerj.10929 (PMC7953866; doi:10.7717/peerj.10929)
Supplement: Supplemental Information 3 [file peerj-09-10929-s003.docx]

Table S2. The similarities of amino acid (aa) sequence of pectin metabolic enzymes of QB4 in PDB database.

Name Description (PDB ID) aa sequence

similarity (%)

Pd1_PL1 Pectate lyase, *Bacillus* sp. N16-5 (3VMV) 58.88

Pd2_PL1 Pectate lyase, Ii *Xanthomonas campestris* (2QX3) 46.19

Pd3_PL1 VexL, a predicted homolog of pectate lyases, *Achromobacter* 46.70

*denitrificans* (6FI2)

Pd4_PL1 Pectinesterase, *Yersinia enterocolitica* subsp. 59.63

*enterocolitica* 8081 (3UW0) Pectate lyase, *Bacillus* sp. N16-5

Pd5_PL1 (3VMV) 54.49

Pd6_PL3 Endo-pectate lyase, *Dickeya chrysanthemi* (3B90) Pectate lyas, 74.56

Pd7_PL10 *Cellvibrio japonicus* (1GXM) 56.37

Pd8_GH105 Unsaturated rhamnogalacturonyl hydrolase (yteR), 49.57

*Bacillus subtilis* (2GH4)

Pd9_GH105 Similar to yteR, *Clostridium acetobutylicum* ATCC 824 (4WU0) 66.77

Pd10_GH105 Similar to yteR, *Clostridium acetobutylicum* ATCC 824 (4WU0) 63.61

Pd11_GH28 Exo-poly-alpha-d-galacturonosidase, *Thermotoga marítima* 65.13

(3JUR)

Pd12_GH28 Polygalacturonase, *Erwinia carotovora* (1BHE) 51.02

Pu1 Deoxy-1-Threo-5-Hexosulose-Uronate Ketol-Isomerase, 65.56

*Enterococcus faecalis* (1YWK)

Pu2 Deoxy-1-Threo-5-Hexosulose-Uronate Ketol-Isomerase, 63.41

*Enterococcus faecalis* (1YWK)

Pu3 4-deoxy-L-threo-5-hexosulose-uronate ketol-isomerase, 69.46

*Escherichia coli* (1X8M)

Pu4 4-deoxy-L-threo-5-hexosulose-uronate ketol-isomerase, 67.74

*Escherichia coli* (1X8M)

Pu5 2-deoxy-D-gluconate 3-dehydrogenase, *Pectobacterium* 66.67

*carotovorum* (4Z9Y)

Pu6 2-deoxy-D-gluconate 3-dehydrogenase, *Pectobacterium* 67.45

*carotovorum* (4Z9Y)

Pu7 2-deoxy-D-gluconate 3-dehydrogenase, *Pectobacterium* 71.20

*carotovorum* (4Z9Y)

Pu8 2-deoxy-D-gluconate 3-dehydrogenase, *Pectobacterium* 71.15

*carotovorum* (4Z9Y)

Pu9 Glucuronate isomerase, *Salmonella enterica* subsp. *enterica* 73.49

serovar Typhimurium (3IAC)

Pu10 Glucuronate isomerase, *Salmonella enterica* subsp. *enterica* 65.16

serovar Typhimurium (3IAC)

Pu11 Uronate isomerase, *Caulobacter vibrioides* CB15 (2Q01) 73.77

Pu12 Fructuronate reductase (UxaA), *Escherichia coli* (4IM7) 42.83

Pu13 Altronate hydrolase, *Shigella flexneri* (3K3S) 71.60

Pu14 Tagaturonate epimerase UxaE, *Cohnella laeviribosi* (6ILA) 44.33

Pu15 Fructuronate reductase (UxaA), *Escherichia coli* (4IM7) 54.68

Pu16 Mannonate dehydratase, *Escherichia coli* K-12 (4EAC) 82.70
