# Supplementary material for: Genes for degradation and utilization of uronic acid-containing polysaccharides of a marine bacterium Catenovulum sp. CCB-QB4
Source: PeerJ. 2021 Mar 9;9:e10929. doi: 10.7717/peerj.10929 (PMC7953866; doi:10.7717/peerj.10929)
Supplement: Supplemental Information 4 [file peerj-09-10929-s004.docx]

Table S3. The similarities of amino acid (aa) sequence of ulvan metabolic enzymes of QB4 in PDB database.

Name Description (PDB ID) aa sequence

similarity (%)

Ud1_PL24 Short ulvan lyase, *Alteromonas* sp. LOR (6BYP) 68.16

Ud2_PL24 Short ulvan lyase, *Alteromonas* sp. LOR (6BYP) 68.32

Ud3_PL24 Short ulvan lyase, *Alteromonas* sp. LOR (6BYP) 76.20

Ud4_PL24 Short ulvan lyase, *Alteromonas* sp. LOR (6BYP) 65.38

Ud5_PL24 Short ulvan lyase, *Alteromonas* sp. LOR (6BYP) 78.53

Ud6_PL24 Short ulvan lyase, *Alteromonas* sp. LOR (6BYP) 90.47

Ud7_PL24 Short ulvan lyase, *Alteromonas* sp. LOR (6BYP) 79.88

Ud8_PL24 Short ulvan lyase, *Alteromonas* sp. LOR (6BYP) 62.04

Ud9_PL24 Short ulvan lyase, *Alteromonas* sp. LOR (6BYP) 63.14

Ud10_PL25 Ulvan lyase-PL25, *Pseudoalteromonas* sp. PLSV (5UAM) 57.83

Ud11_PL25 Ulvan lyase-PL25, *Pseudoalteromonas* sp. PLSV (5UAM) 57.25

Ud12_PL25 Ulvan lyase-PL25, *Pseudoalteromonas* sp. PLSV (5UAM) 74.67

Ud13_PL25 Ulvan lyase-PL25, *Pseudoalteromonas* sp. PLSV (5UAM) 66.14

Ud14_PL40 No significant similarity found

Ud15_GH105 Unsaturated 3s-rhamnoglycuronyl Hydrolase, *Nonlabens* 48.41

*ulvanivorans* (4CE7)

Ud16_GH105 Unsaturated 3s-rhamnoglycuronyl Hydrolase, *Nonlabens* 52.74

*ulvanivorans* (4CE7)

Ud17_GH105 Unsaturated 3s-rhamnoglycuronyl Hydrolase, *Nonlabens* 56.33

*ulvanivorans* (4CE7)

Ud18_GH105 Unsaturated 3s-rhamnoglycuronyl Hydrolase, *Nonlabens* 51.62

*ulvanivorans* (4CE7)

Ud19_GH105 Unsaturated 3s-rhamnoglycuronyl Hydrolase, *Nonlabens* 51.79

*ulvanivorans* (4CE7)

Ud20_GH78 Rhamnosidase B, *Bacillus* sp. GL1 (2OKX) 36.60

Ud21_GH78 Alpha-rhamnosidase, *Dictyoglomus thermophilum* H-6-12 (6I60) 47.51

Ud22_GH78 Putative rhamnosidase, *Streptomyces avermitilis* MA-4680 54.13

(3W5M)

Ud23_GH78 Putative Alpha-Rhamnosidase, *Bacteroides thetaiotaomicron* 39.97

(3CIH)

Ud24_GH78 Putative rhamnosidase, *Streptomyces avermitilis* MA-4680 56.81

(3W5M)

Ud25_GH43 Glycoside hydrolases, *Zobellia galactanivorans* (4U6D) 50.00

Ud26_GH43 Endo-1,4-beta-xylanase, *Bacteroides thetaiotaomicron* VPI-5482 43.61

(3KST)

Ud27_GH43 Beta-xylosidase, *Geobacillus thermoleovorans* (5Z5D) 54.23

Ud28_GH43 Beta-xylosidase, *Geobacillus thermoleovorans* (5Z5D) 45.62

Ud29_GH43 Glycoside hydrolase family 43, *Halothermothrix orenii* H 168 45.75

(4QQS)

Ud30_GH43 Endo-1,4-beta-xylanase D, *Bacteroides thetaiotaomicron* (3QZ4) 49.20

Ud31_GH43 Glycoside hydrolase family 43, *Halothermothrix orenii* H 168 39.10

(4QQS)

Ulu1 Sulfatase, *Pseudoalteromonas fuliginea* (6BIA) 37.97

Ulu2 Exo-4S-kappa carrageenan S1 sulfatase, *Pseudoalteromonas* 44.92

*fuliginea* (6PRM)

Ulu3 L-rhamnose Mutarotase, *Rhizobium leguminosarum* (2QLX) 67.65

Ulu4 L-rhamnose Isomerase, *Pseudomonas stutzeri* (2HCV) 79.24

Ulu5 Carbohydrate Kinase, *Novosphingobium aromaticivorans* 46.17

DSM 12444 (3H6E)

Ulu6 Phosphate Aldolase, *Streptococcus pneumoniae* TIGR4 (4C25) 53.15

Ulu7 Aldehyde dehydrogenase A, *Vibrio variabilis* (6J75) 72.82

Ulu8 (S)-mandelate dehydrogenase, *Pseudomonas putida* (6BFG) 57.53

Ulu9 Xylose isomerase, *Piromyces* sp. E2 (5NH4) 72.67

Ulu10 Xylulokinase, *Brucella ovis* ATCC 25840 (5VM1) 46.37

Ulu11 Xylose Operon Regulatory Protein, XylR, *Escherichia coli* K-12 60.98

(4FE4)
