# Supplementary material for: Genes for degradation and utilization of uronic acid-containing polysaccharides of a marine bacterium Catenovulum sp. CCB-QB4
Source: PeerJ. 2021 Mar 9;9:e10929. doi: 10.7717/peerj.10929 (PMC7953866; doi:10.7717/peerj.10929)
Supplement: Supplemental Information 5 [file peerj-09-10929-s005.docx]

Table S4. The similarities of amino acid (aa) sequence of ulvan metabolic enzymes of QB4 in PDB database.

Name Description (PDB ID) aa sequence

similarity (%)

Gd1_GH1 β-glucosidase A, *Hungateiclostridium thermocellum* 63.23

(5OGZ) ATCC 27405

Gu1 Glucokinase (Glk), *Escherichia coli* (1Q18) 66.56

Gu2 Glucose-6-phosphate Isomerase, *Escherichia coli* (3NBU) 71.80
