# Supplementary material for: Genes for degradation and utilization of uronic acid-containing polysaccharides of a marine bacterium Catenovulum sp. CCB-QB4
Source: PeerJ. 2021 Mar 9;9:e10929. doi: 10.7717/peerj.10929 (PMC7953866; doi:10.7717/peerj.10929)
Supplement: Supplemental Information 6 [file peerj-09-10929-s006.docx]

Table S5. List of bacterial species possessing 5 or more ulvan lyases in CAZy database.

Strains name Number of

PL24s PL25s PL28s PL37s PL40s

*Alteromonas mediterranea* AitCH17 3 2

*A. mediterranea* 76-1 3 2

*Catenovulum agarivorans* 5

*C. agarivorans* DS-2 4 3

*Catenovulum* sp. CCB-QB4 9 4 1

*Formosa agariphila* KMM3901 1 1 3

*Tamlana* sp. UJ94 1 1 2 1 2

*Polaribacter* sp. BM10 2 1 4

Seonamhaeicola sp. S2-3 2 2 2

*Siansivirga zeaxanthinifaciens* CC-SAMT-1 1 3 2 2

*Wenyingzhunangia fucanilytica* CZ1127 1 1 5
